# HRCTCov19-a high-resolution chest CT scan image dataset for COVID-19 diagnosis and differentiation

**DOI:** 10.1186/s13104-024-06693-z

**Published:** 2024-01-22

**Authors:** Iraj Abedi, Mahsa Vali, Bentolhoda Otroshi, Maryam Zamanian, Hamidreza Bolhasani

**Affiliations:** 1 Department of Medical Physics, School of Medicine, Isfahan University of Medical Sciences, Isfahan, Iran; 2 Department of Electrical and Computer Engineering, Isfahan University of Technology, Isfahan, Iran; 3 Department of Radiology, School of Medicine, Arak University of Medical Sciences, Arak, Iran; 4 Department of Computer Engineering, Science and Research Branch, Islamic Azad University, Tehran, Iran

**Keywords:** COVID-19, CT scan, Computed tomography, Chest image, Dataset, Medical imaging

## Abstract

**Introduction:**

Computed tomography (CT) was a widely used diagnostic technique for COVID-19 during the pandemic. High-Resolution Computed Tomography (HRCT), is a type of computed tomography that enhances image resolution through the utilization of advanced methods. Due to privacy concerns, publicly available COVID-19 CT image datasets are incredibly tough to come by, leading to it being challenging to research and create AI-powered COVID-19 diagnostic algorithms based on CT images.

**Data description:**

To address this issue, we created HRCTCov19, a new COVID-19 high-resolution chest CT scan image collection that includes not only COVID-19 cases of Ground Glass Opacity (GGO), Crazy Paving, and Air Space Consolidation but also CT images of cases with negative COVID-19. The HRCTCov19 dataset, which includes slice-level and patient-level labeling, has the potential to assist in COVID-19 research, in particular for diagnosis and a distinction using AI algorithms, machine learning, and deep learning methods. This dataset, which can be accessed through the web at http://databiox.com, includes 181,106 chest HRCT images from 395 patients labeled as GGO, Crazy Paving, Air Space Consolidation, and Negative.

## Introduction

Coronavirus disease 2019 (COVID-19) is a highly contagious disease that causes severe respiratory distress syndrome. Since 2019, COVID-19 has spread fast throughout several cities in China and other nations [1]. According to the WHO, as of February 2022, there have been over 378,000,000 confirmed cases worldwide, with over 5,670,000 fatalities. There are two main methods for determining whether a person is infected with COVID-19 or not: the first uses chest computerized tomography (CT) images, and the second uses a reverse-transcription polymerase chain reaction (RT-PCR) test, which is based on a patient’s respiratory samples, such as nasal mucus [2, 3]. Although the gold standard for verifying COVID-19 mostly depends on microbiological tests like real-time polymerase chain reaction (RT-PCR) [4], radiological imaging, particularly thin-section CT, is critical. CT is important for the care and follow-up of patients with COVID-19 since it ensures timely treatment and prevents the illness from spreading to other potentially infected persons. It also aids in the assessment of respiratory morbidity [4, 5].

High-resolution CT (HRCT) of the chest is now routinely employed not just in the diagnosis of COVID-19 pneumonia, but also in the monitoring of its course. In the diagnosis of COVID-19 pneumonia, HRCT chest has a sensitivity of 56–98%. Ground Glass Opacity (GGO) is the most prevalent finding on chest CT in patients with COVID-19 pneumonia and although is a nonspecific finding, when seen in a typical pattern, is suggestive of COVID-19 pneumonia [6, 7].

Typical imaging findings are peripheral bilateral GGO with or without consolidation or Crazy-Paving pattern, reverse halo signs or other organizing pneumonia (OP)-related findings, and multifocal GGO of the rounded morphology with or without consolidation or visible intralobular lines (Crazy-Paving) [8].

Atypical characteristics include lobar or segmental consolidation in bacterial pneumonia, cavitation in necrotizing pneumonia, and tree-in-bud opacities with centrilobular nodules, which may be seen in a variety of community-acquired infections and aspiration [9].

During the COVID-19 pandemic, radiologists have conducted much research for fast analysis of a huge number of CT images. To overcome these difficulties, many researchers (such as [10, 11]) have established in-depth learning techniques for COVID-19 filtering from CTs. While the findings of this study are quite positive, there are some limitations. Publicly accessible datasets often comprise a small number of individuals and exclude other types of respiratory illnesses to allow for comparison. Additionally, given the present state of medical professionals’ involvement in the treatment of COVID-19 patients, it is improbable that they will have time to gather and interpret the COVID-19 CT scan information. Additionally, CT scans might originate from a variety of different sources and be acquired using a variety of different imaging methods, limiting the breadth of an integrated analysis [12–14].

Today, no one is unaware of artificial intelligence’s potential in all fields. However, the main difficulty and necessity for the deployment of artificial intelligence-based algorithms is the availability of a vast database of information sufficient to bring artificial intelligence’s performance to an acceptable level. Access to huge data sets is problematic in the medical field owing to the high cost of data collecting, patient privacy concerns, and so on. Transfer learning is used to accomplish this. For this purpose, artificial intelligence is taught on a large database and then applied to a smaller database using the trained model. This study’s objective is to create a dataset of CT scan images of COVID-19 patients. It is envisaged that academics will utilize the provided dataset in the future to utilize in pre-trained algorithms.

### Data description

**Fig. 1:**
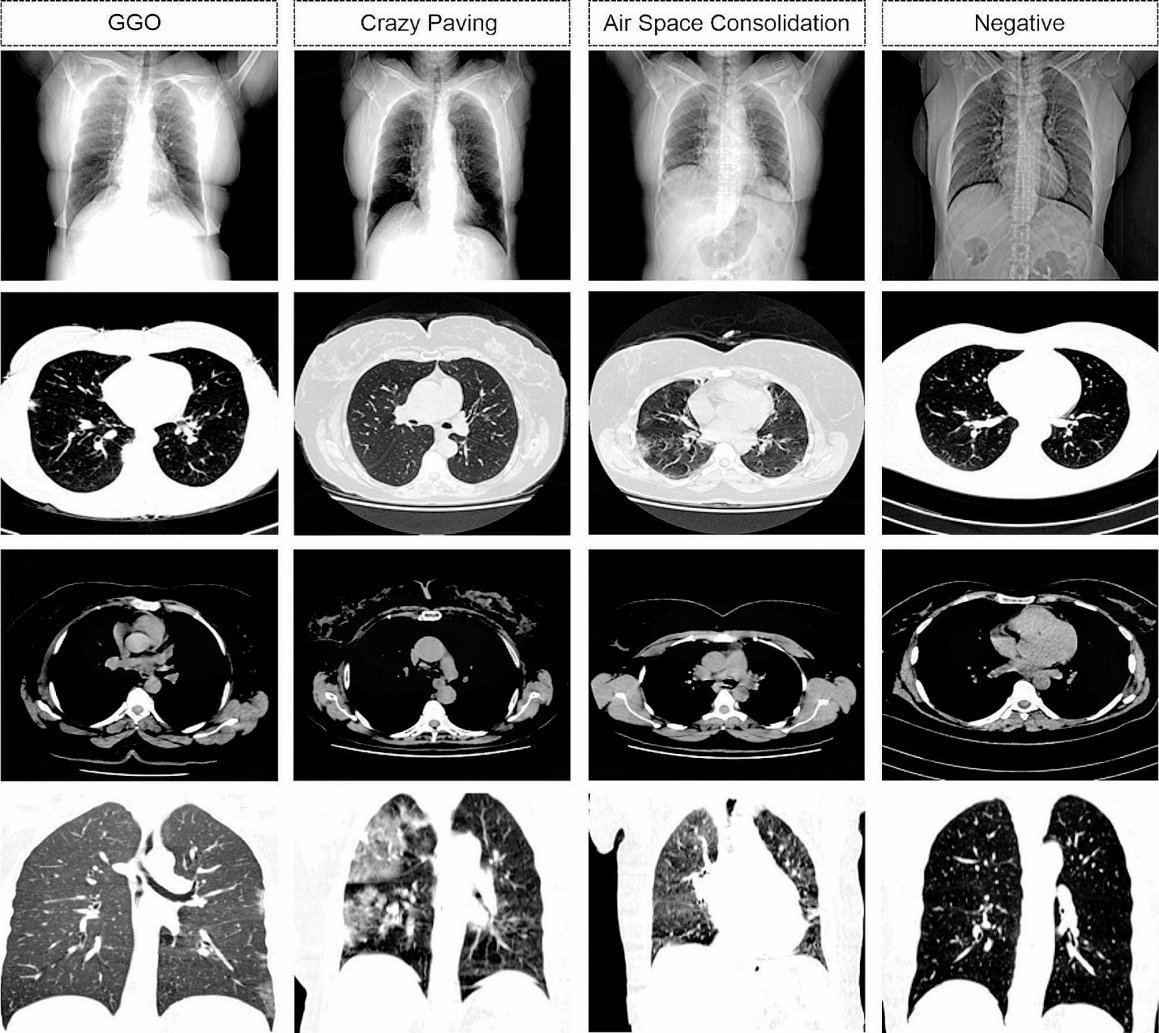
HRCTCov19 image dataset samples from each four labels

The main idea behind the organization of this dataset comes from a 2020 study completed and published by DataBioX researchers on the histopathology imaging dataset for grading breast invasive ductal carcinoma [15]. The HRCTCov19 dataset includes 181,106 high-resolution grayscale chest pictures from 395 patients, of which 197 were female, 193 were male, and 5 were not defined. The patients were categorized into four categories: GGO (*N* = 288), Crazy Paving (*N* = 57), Air Space Consolidation (*N* = 27), and Negative (*N* = 23). Some samples from each category and a summary of this dataset specification are represented in Fig. 1; Table 1, respectively.

**Table 1:** A summary of HRCTCov19 dataset specification

Label	Name of data file/data set	File types (file extension)	Identifier (DOI or accession number)
Data file	01_HRCTCov19_Dataset.xlsx	Excel file (.xlsx)	Zenodo data (10.5281/zenodo.10252424)(16)
Data set 1	Air Space Consolidation.rar	RAR file (.rar)	Zenodo data (10.5281/zenodo.10252424)(16)
Data set 2	Crazy Paving.rar	RAR file (.rar)	Zenodo data (10.5281/zenodo.10252424)(16)
Data set 3	GGO.rar	RAR file (.rar)	Zenodo data (10.5281/zenodo.10252424)(16)
Data set 4	Negative.rar	RAR file (.rar)	Zenodo data (10.5281/zenodo.10252424)(16)

This dataset was compiled from emergency room visits at Milad hospitals between February and September of 2021. It consists of people who got a chest CT and an RT-PCR for suspected COVID-19 pneumonia.

All CT examinations were performed with a 128-row Multidetector CT system (SOMATOM Definition Flash, Siemens Healthcare system, Germany). Scanning coverage was from the thoracic inlet to the inferior level of the costophrenic angle. The CT scanning protocol was as follows: tube voltage of 100KV, tube current of 100–200 mA (automatic exposure control employed), rotation time of 0.35 s, pitch of 1.4 mm, detector collimation of 0.6 mm, slice thickness/reconstruction thickness of 5 mm/1 mm. All scans were performed in the supine position during end-inspiration.

**Fig. 2:**
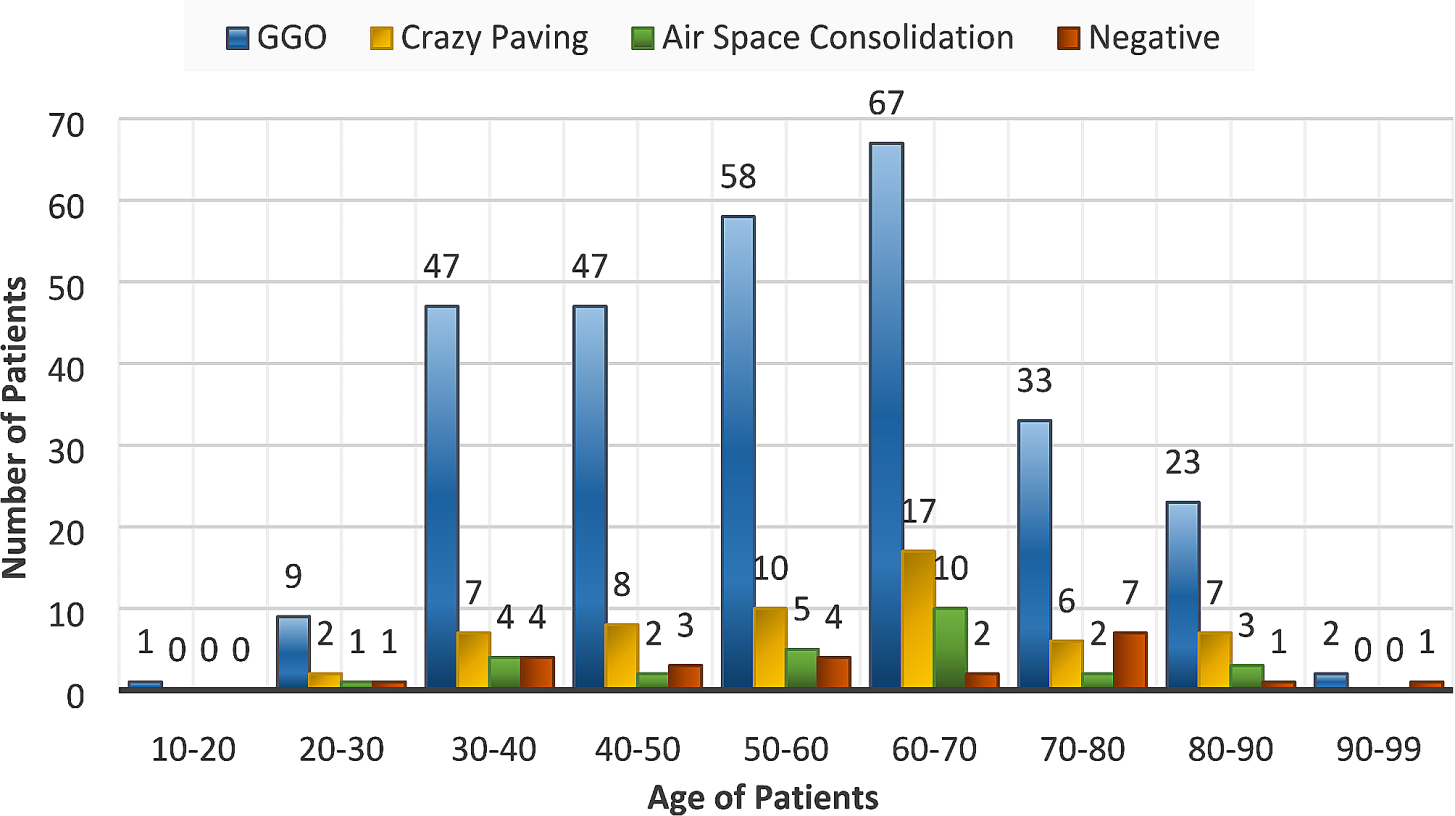
Distribution of cases based on their age and infection type in the dataset

Data were transferred to the image processing workstation. These CT images were reviewed on the above-mentioned workstation at the lung window (width of 1000 HU and window level of − 700 HU). To ensure the accuracy of the analysis, all images were independently evaluated by an experienced radiologist. The diagnosis of Covid illness was further confirmed by the results of the patients’ RT-PCR tests. Figure 2 Shows information about the number of patients with four infections in the dataset.

The COVID dataset presented in other studies has either provided CT data just from COVID patients alone [16], or the data presented falls into two categories: COVID and Normal [13, 17–19].

In a study by Shakuri et al., an open-source repository of more than 1,000 CT scans of COVID-19 lung infection was published and developed by authorized radiologists in the study. CT scans were obtained between March 2020 and January 2021 from two main university hospitals located in Mashhad. Comparative assays, such as RT-PCR and associated clinical signs, were used to confirm COVID-19 infections. The authors state that all data is recorded in the DICOM standard as 16-bit images on a gray scale of 512 × 512 pixels [20].

Afshar, et al. introduced the COVID-CT-MD dataset which consists of 169 confirmed positive COVID-19 cases, 76 normal cases, and 60 community-acquired pneumonia (CAP) cases. All these cases were collected from the Babak Imaging Center in Tehran, Iran [12].

Zaffino et al. published a dataset including 50 COVID-19-positive patients in ITK-based file format as a non-contrast chest CT. The presented datasets were obtained from Azienda Ospedaliera Pugliese-Ciaccio in Catanzaro, Italy by two different scanners [21].

In addition, Soares et al. provided a COVID-CT scan dataset from the Public Hospital of the Government Employees of Sao Paulo Metropolitan Hospital of Lapa in Brazil. This dataset includes 210 patients, only 80 of them are infected with COVID-19 and collected from March 15 to June 15, 2020 year. The matrix size of the images was 512 × 512 with breath-hold at full inspiration [22].

The database we provide contains HRCT images. A CT scan of the chest uses X-rays to obtain images of the lung tissue. The images are obtained in “slices,” or thin views, that are put together to form an image. The slices of an HRCT are much thinner than with a standard CT scan, giving a more detailed image. HRCT scans are performed in one-millimeter slices [23]. Given the variety of infection patterns covered in this large dataset, it may be used as a starting point for more extensive data-train-driven AI models.

### Limitations

The limitations we faced while preparing the dataset were missing or incorrect data, as well as low image quality due to respiration artifacts caused by the patient’s difficulty breathing. Some categories, such as “Crazy Paving” and “Air Space Consolidation,” have less data than the GGO category. The use of only one radiologist as an expert and the other for the sample population as images were taken from only one hospital.

## Data Availability

The datasets generated during and/or analyzed during the current study are available in the Zenodo repository (10.5281/zenodo.10252424). The latest version of the data can also be accessible at https://databiox.com, the DataBioX website.

## Abbreviations

HRCT: High-resolution CT
GGO: Ground Glass Opacity
OP: Organizing pneumonia
